# Behaviour-directed interventions for problematic person transfer situations in two dementia care dyads: a single-case design study

**DOI:** 10.1186/s12877-022-02952-5

**Published:** 2022-03-29

**Authors:** Hanna Lagerlund, Charlotta Thunborg, Maria Sandborgh

**Affiliations:** 1 Rehabilitation Unit, Nyköping Municipality, 611 83 Nyköping, Sweden; 2 Department of Neurobiology, Care Sciences and Society Division of Clinical Geriatrics, Karolinska Institute, Karolinska vägen 37A, 171 76 Stockholm, SE Sweden; 3 School of Health, Care and Social Welfare, Mälardalen University, Box 883, 721 23 Västerås, Sweden

**Keywords:** Person transfer situation, Single-case design, Functional behaviour analysis, Dementia, Special care unit, Physiotherapy

## Abstract

**Background:**

Persons with dementia living in nursing homes need assistance with moving and transfers; however, caregivers assisting persons with dementia in their daily person transfers report strain-related and complicated transfer-related behavioural problems. The reciprocity of complex dyadic transfer-related behaviours is affected by environmental factors, the health status of the person with dementia and the caregiver’s skills and knowledge. The aim of this study was to explore tailored interventions guided by a functional behaviour analysis for problematic person transfer situations in two dementia care dyads.

**Methods:**

This study was a quasi-experimental single-case study with an A-B design. Tailored interventions were developed in a five-step model for functional behavioural analysis. The study was conducted in a dementia special care unit at a nursing home, and the inclusion criteria were caregivers’ experiences of physical strain and/or resistiveness to care, which led to complex transfer-related behaviour. Two care dyads were included. Transfer situations were video-recorded and evaluated with the Dyadic Interaction in Dementia Transfer Assessment Scale, Pain Assessment in Advanced Dementia Scale, and Resistiveness to Care Scale for Dementia of the Alzheimer’s Type. The caregiver experience was evaluated with study-specific items addressing caregiver self-efficacy, catastrophizing thoughts, perceived control, and perceived physical strain. Scorings were graphically displayed. The graphs were inspected visually to identify changes in trend, level, latency, and variability. Nonoverlap of all pairs (NAP), including 90% confidence intervals (CIs), was calculated to complement the visual inspection.

**Results:**

Verbal and nonverbal discomfort decreased in care dyad 1, which mirrored the caregiver changes in adapting their actions to the needs of the person with dementia. High variability was seen in both the intervention and the baseline phases in care dyad 2. In both care dyads, caregiver transfer-related behaviour improved.

**Conclusions:**

The results indicate that the transfer-related behaviours of the care dyad might be improved through a behaviour-directed intervention tailored to meet the care dyad´s needs. The small number of cases and observations limits the generalizability, and the results should be interpreted in consideration of the piloting approach of the study.

**Supplementary Information:**

The online version contains supplementary material available at 10.1186/s12877-022-02952-5.

## Introduction

Persons with dementia (PwD) living in nursing homes and dementia special care units (SCUs) are often vulnerable individuals with complex and advanced needs [1]. Dementia reduces cognitive function and impairs walking and balance, which leads to mobility problems and is therefore a common cause of dependency that affects daily living activities [2, 3]. People in the late stages of dementia living in SCUs require increasing support over time from caregivers [4]. Due to cognitive impairment and diminished consent capacity of persons in the late stages of dementia, they risk not being included in research that aims to address daily difficulties, such as person transfer situations. Despite these ethical challenges, it is important to study how to improve person transfer situations, which occur frequently and greatly affect the quality of life for a PwD [5].

A transfer situation in an SCU is often constructed of one or two caregivers helping a PwD move, such as from sitting to standing or transferring from a bed to a wheelchair. A transfer situation is often complicated and influenced by environmental, psychological, and physical factors and could be seen as a complex care dyadic behaviour involving both the PwD and the professional caregiver [6].

A care dyad consists of two people: one caregiver and one care receiver [7]. Most research addressing dementia care dyads concerns a PwD and an informal caregiver [8, 9]. Caring for a PwD is sometimes challenging, and the professional caregiver has a significant impact on the quality of life and the outcomes when supporting optimal functions in the PwD [10]. What caregivers do and the way they do it affects the PwD and influences the PwD’s behaviour in a caring situation [11, 12]. One example is the resistiveness to care [13], which can also be referred to as a transfer situation [6]. If a PwD displays resistiveness to care by striving backwards, for example, the caregivers might experience physical strain [14], which in turn can result in uncompleted transfers and increased reciprocal struggling by the care dyad [15].

The reciprocal relationship between the members of the care dyad, their interrelated behaviour, the functional status and overall health of the PwD and the caregivers’ competence and flexibility alongside the environmental factors (e.g., walking and moving aids, and a stressful working environment) could be interpreted as a dynamic triad within Social Cognitive Theory (SCT) [16]. In the dynamic triad behavior is conceptualized as a person's skills and actions, the environment is a person's social and physical surroundings, and individual factors are a persons beliefs, cognitive abilities and physical characteristics. In the transfer situation all three systems interact with each other; therefore, a change in one will influence the others as well [16].

There is a knowledge gap concerning the complexity of caring for a PwD in transfer situations, especially with regard to interventions that address the reciprocity of the care dyad, environment, and behaviour. In a systematic review inconclusive, but promising evidence was found for the efficacy of functional analysis-guided interventions for challenging behaviour in dementia [17]. Physiotherapy interventions that consider the complexity of dyadic reciprocity in functional behavioural analyses (FBAs) could provide a better understanding of the function of the care dyad´s transfer-related behaviour, which in turn could facilitate more effective tailored interventions for problematic dyadic behaviour [18], e.g., dyadic transfer-related behaviour, as demonstrated in a single case study (SCS) by Thunborg and colleagues [6]. However, systematic and clinical replications of an SCS are needed to increase the generalizability of the findings [19].

The aim of this study was to explore tailored interventions guided by an FBA for problematic person transfer situations in two dementia care dyads.

## Methods

### Design

In this study, a quasi-experimental single-case design was used, with a baseline phase (A) and an intervention phase (B), and tailored interventions were developed. The key characteristics of an SCS are ongoing assessment, baseline assessment, performance stability and different phases [19]. The design is clinically relevant and features the opportunity to meet the complexity of person transfer-related behavioural problems in care dyads by tailoring and modifying interventions according to the care dyad’s changing needs. This study is a replication of a former study by Thunborg et al. [6]; however, it was implemented in another setting, and with a small number of cases constitutes a piloting approach.

### Setting and participants

The study was conducted in an SCU with 19 PwDs and 13 caregivers giving constant care and support. Caregivers were either licenced practical nurses or nursing assistants. Two care dyads, i.e., the PwD and the caregivers assisting in the person transfer situation, were included. The first author conducted the recruitment of the care dyads in collaboration with the caregivers, the manager of the care unit and the responsible nurse. The inclusion criteria for the care dyad were that caregivers had reported experience of physical strain when assisting the PwD and/or that the PwD displayed resistiveness to care, which led to difficulties for the PwD in performing any of the following person transfer situations: lying to sitting, sitting to standing, walking, standing to sitting or sitting to lying. PwD who met the selection criteria but were expected to be at risk of being psychologically or physically adversely affected by participating or who were assessed to be in or near a late palliative phase in life were excluded.

All professional caregivers in the SCU were asked to participate. In total, eight gave their written consent to participate. All who consented were included in the study, and they were video recorded at least two times. As all caregivers worked shifts on different schemas, maintaining the same caregiver who was supporting the PwD throughout all video-recorded transfer situations was not possible. A care dyad is therefore defined as a PwD and the caregivers who assist in a given person transfer situation. For participant characteristics, see Table 1.

**Table 1:** Care dyad characteristics

Care dyad	Gender	Age	Diagnosis	Time in Special Care Unit
1	woman	83	Alzheimer´s disease	2 years
2	woman	78	Alzheimer´s disease	2 years
**Care dyad**	**Gender**	**Age**	**Professional role**	**Years working as a caregiver**
1	woman (*N* = 4)	37–58 years	Licensed practical nurses (*N* = 4)	2–29
2	woman (*N* = 4)	20–51 years	Licensed practical nurses (*N* = 3); nursing assistant (*N* = 1)	2–25

### Data collection

Data were collected between April and July 2019. One problematic person transfer situation for each care dyad was identified through semistructured interviews with the caregivers concerning the character of the problematic transfer situation, affecting factors and the functional ability of the PwD. In total, 23 person transfer situations were video-recorded and analysed (see Table 2). The time required for each person transfer situation was measured with a digital timer by observing and calculating the time spent from start to finish. The tenets of SCT and FBA guided the choice of data to be collected.

**Table 2:** Number of video-recorded observations and length of phases

	**Number of video-recorded observations**		**Length of phases in days**	
	Baseline (A)	Intervention (B)	Baseline (A)	Intervention (B)
Care dyad 1	6	6	18	29
Care dyad 2	5	6	8	36

### Dyadic interaction in dementia transfer assessment scale

To assess problematic person transfer situations, the Dyadic Interaction in Dementia Transfer Assessment Scale (DIDTAS) [20] was used. Development of DIDTAS was guided by the features of SCT. It is an observational assessment scale to evaluate problematic person transfer situations when a caregiver assists a PwD in a person transfer situation. The scale contains 17 items: items 1–8 assess the actions of the PwD, and items 9–17 assess the actions of the caregiver. A higher score indicates a more difficult person transfer situation. The intraclass correlation (ICC), which represents the reliability for each of the 17 items, ranges from 0.34 to 0.92 for interrater reliability and from 0.56 to 0.92 for intrarater reliability [21]. See additional file 1 DIDTAS.

### Pain assessment in advanced dementia scale

To assess pain during the person transfer situation for the PwD, the Pain Assessment in Advanced Dementia Scale (PAINAID) [22] was used. The PANAID was developed to assess pain in older cognitively impaired individuals who cannot self-report their pain experience. The PAINAD includes five items related to five behaviours. The items include breathing, negative sounds (e.g., crying and moaning), facial expressions, body language and consolability, which are rated from 0 to 2 and summarized based on a total score of 0 to 10. A possible interpretation of the scores is as follows: 1–3 = mild pain; 4–6 = moderate pain; and 7–10 = severe pain. PAINAID shows good conceptual validity and satisfactory interrater validity [22].

### Resistiveness to care scale-dementia alzheimer type

Resistiveness to care was evaluated with the Resistiveness to Care Scale-Dementia Alzheimer Type (RTC-DAT) [23], which consists of 13 items concerning the behaviours: turn away, pull away, push away, push/pull, grab object, grab person, adduct, hit/kick, say no, cry, threaten, scream/yell and clench mouth. Each behaviour is observed and rated based on prevalence and severity. RTC-DAT gives a score between 0 and 156, with a lower score representing a small degree of resistiveness to care. Regarding reliability, internal consistency estimates from 0.82–0.87 and good to excellent kappa values have been demonstrated [23].

### Caregiver´s self-reported ratings

Before each observation, the caregiver completed self-reported ratings regarding their self-efficacy, catastrophizing thoughts and perceived control for the transfer situation in question. Study specific questions were developed by using items from existing instruments to ensure correct wording of questions. These questions were used based on their relevance for the care dyads’ problems.

Four items addressing the caregivers´ self-efficacy for transfer- related behaviours were developed in the context of SCT [24] and were formulated according to Albert Bandura’s ‘Guide for constructing self-efficacy scales’ [25]. Three items were adapted from the Coping Strategies Questionnaire (CSQ) and addressed one statement measuring catastrophizing thoughts and two questions measuring perceived control [26]. The item perceived physical strain was adapted from the Patient Transfer Assessment Instrument (PTAI) and measured after the observation with the following question: “How do you experience physical strain during the person transfer situation (0 = not physically strenuous at all, 10 = physically very strenuous)” [27]. For further details, see additional file 2.

### Procedural steps in the FBA model

**Fig. 1:**
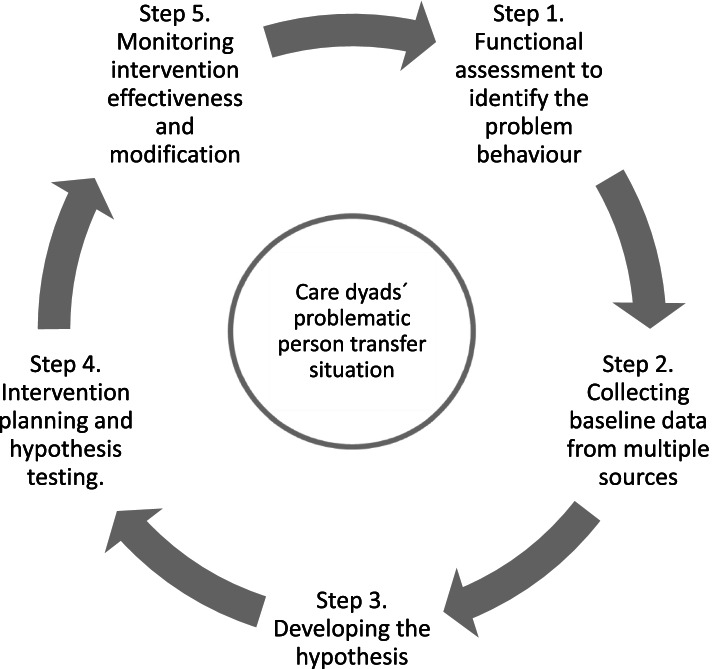
Five-step model for the functional behavioural analyses (FBAs), adapted from Thunborg et al. (6, p. 3)

We used a five step FBA-model (see Fig. 1). During the first step, cognitive, functional and/or environmental factors associated with the occurrence (and nonoccurrence) of specific problematic transfer-related behaviour were identified for each care dyad. Sources for information on the person transfer situation were caregiver reports, medical records and next-of-kin information. The first author observed the person transfer situation without video recording and performed a general physiotherapy assessment (e.g., range of motion, any signs of pain, anxiety and communication difficulties). During the baseline phase (step 2), the caregivers were instructed to administer care as usual when assisting the PwD in the transfer situation. The transfer situation was video-recorded six (care dyad 1) and five (care dyad 2) times by the first author. The video-recorded environment-related factors, antecedents for the target behaviour, the behaviour itself and the consequences of the behaviour were analysed and rated by all authors. After gathering the baseline data, the DIDTAS items of interest were chosen alongside the ratings from the RTC-DAT, PAINAID and caregivers’ self-reports, and a problematic transfer-related target behaviour was identified. In step 3, a hypothesis based upon the assessment in steps 1 and 2 describing the target behaviour in sufficient detail was developed. In step 4, the hypothesis was tested by implementing one tailored intervention in each care dyad. In step 5, the intervention effectiveness was monitored, i.e., the caregivers provided rating on the self-report assessment scales, and the person transfers were video-recorded. The caregivers were instructed to administer care according to the tailored intervention when assisting the PwD in the transfer situation. The transfer situation was video-recorded six times in step 5 in each care dyad.

### Dyad characteristics and baseline data

#### Care dyad 1

##### Step 1. Functional assessment to identify the problem behaviour

The PwD in care dyad 1 was an 83-year-old woman with Alzheimer´s disease. She had suffered from neck disabilities of unknown origin most of her adult life and had fibromyalgia since the age of 50. In recent years, she had unspecified back pain and general tremor. She had paratonia in both the upper and lower limbs, and an assessment with the Paratonia Assessment Instrument (PAI) [28] indicated moderate paratonia (value = 2) on a scale from 0- 5, which resulted in involuntary resistance in passive movements. She rarely took the initiative for speech but was able to answer simple spoken questions and was judged by the caregivers to be able to understand the meaning of instructions and conversations to some extent. She was unable to perform a cognitive test; therefore, her cognitive ability was uncertain. The caregivers supposed she had adequate hearing and vision. She used a mobile lift in all daily transfers to the wheelchair. Two caregivers on each side of the PwDs bed took part in the morning care routine when the lifting sling was applied. The caregivers reported that her entire body became tense and she cried out and was perceived to be struggling against the caregivers when they turned her from side-to-side with the help of sliding and draw sheets to apply the lifting sling. The caregivers experienced the transfer situation as difficult and physically heavy, and they also inferred that she did not feel well during the transfer situation. The transfer situation proceeded with other similar transfer situations when changing incontinence protection.

##### Step 2. Collecting baseline data from multiple sources

During baseline (A), the problem behaviour was best described by the high ratings of DIDTAS items 6 and 7, indicating that the PwD expressed verbal and non-verbal discomfort. The high caregiver ratings of DIDTAS items 9 and 10 indicated a lack of caregiver instructions before the beginning of the transfer and a lack of a clear verbal commands about the transfer. The high ratings of DIDTAS items 11 and 14 indicated that the caregivers did not wait for the PwD to respond during the transfer and that the caregivers did not adapt their actions to facilitate the PwD in the person transfer situation. See appendix DIDTAS. Information about the caregiver’s experiences of physical strain was provided through self-reports. The PAINAID (score = 0) and RTC-DAT (score = 0) values indicated that the PwD had no pain and did not display any resistiveness to care in the person transfer situation.

##### Step 3. Developing the hypothesis

The hypothesis suggested that when the PwD experienced a change in position for which she was unprepared, her unease and paratonia increased, which was expressed through grabbing objects and shouting, and that motor protective reflexes were triggered in combination, with increased muscle tension over the whole body. The caregivers experienced the transfer situation as physically heavy and expressed difficulties in consoling the PwD. Eye contact, maintaining physical contact and clear verbal instructions seamed to decrease anxiety and paratonia. Rapidness during the transfer situation seemed to increase unease and paratonia. The behavioural goals were therefore to decrease the verbal and non-verbal discomfort expressed by the PwD (DIDTAS items 6 and 7) and to increase the adaptation to the PwD during patient transfer by the caregiver (DIDTAS items 9, 10, 11 and 14).

#### Care dyad 2

##### Step 1. Functional assessment and problem behaviour identification

The PwD in care dyad 2 was a 78-year-old woman with Alzheimer´s disease and no other known diseases. She had severe difficulties sitting down on the toilet, especially in the mornings. When the caregivers assisted physically, she could react with resistiveness and strive in the opposite direction. The nursing staff found that their verbal instructions did not reach the women. The person transfers worked best when she took the initiative herself to step out of bed, but even on these occasions, she could experience problems sitting down on the toilet. The caregivers noticed that when two caregivers assisted the person transfer, the interaction with her was impaired. Therefore, only one caregiver assisted. She had impaired vision and used glasses, although she seldom wore them when she got up from bed and walked to the toilet during the morning routine. The caregivers supposed she had adequate hearing. She communicated with few words and seldom provided adequate responses to direct questions. The caregivers thought she was able to understand the meaning of instructions and conversations to some extent, although the linguistic understanding was difficult to assess. The physiotherapy assessment showed that she had sufficient muscle strength and balance to sit down on the toilet.

##### Step 2. Collecting baseline data from multiple sources

During baseline (A), the problem behaviour was best described by the high ratings of DIDTAS item 1, indicating that the PwD was not able to remain attentive during the person transfer situation. The high ratings of DIDTAS item 14 indicated that the caregivers did not adapt their actions to facilitate the PwD in the person transfer situation. Important information for the FBA was also obtained from the PwD´s RTC-DAT scores, indicating that the PwD showed resistiveness to care by grabbing the toilet handle and walking away from the caregiver in the transfer situation. A PAINAID score of 0 indicated that the PwD had no pain during the person transfer situation.

##### Step 3. Developing the hypothesis

The hypothesis was that when the PwD was assisted physically and asked verbally to sit down on the toilet, she had severe difficulties interpreting the instructions and understanding the goal with the person transfer. Verbal instructions could lead to compliance, no action at all or walking away from the toilet. When the caregiver assisted physically, she often reacted with resistiveness and grabbed the toilet handles and did not release them. The caregivers adjusted their instructions, e.g., combined them with visual and auditive cues or used a diverting step, e.g., making the bed and then going back to the toilet. The PwD seemed to follow the instructions better if the caregiver was standing on her right side instead of in front or on her left side. Since the PwD often forgot to use her glasses, she might experience a reduced ability to interpret visual cues. The behavioural goals were therefore to increase the PwD’s attentiveness (DIDTAS item 1) and to increase the caregiver’s adaption to the PwD in the transfer situation (DIDTAS item 14).

### Data management and analysis

The scores for the outcome measures were displayed graphically, and medians for each outcome and phase were calculated. The graphed data points, each representing a person transfer observation, were drawn and connected with trend lines over time and within a given phase. The graphs were inspected visually to identify changes in trend, level, latency and variability [19]. Nonoverlap of all pairs (NAP), including 90% confidence intervals (CIs), was calculated to complement the visual inspection. NAP is a nonparametric technique for measuring “the percent of nonoverlapping data between baseline and treatment phases” (Parker I. & Vannest, 2009, p 359). The NAP value is equal to the empirical area under the curve (AUC) of a receiver operating characteristic test [29]. To fit the analysis with the NAP, the item scoring for DIDTAS was reversed when calculating the NAP.

Statistical analysis with the Mann–Whitney U-test was used to analyse the time required from start to finish for each person transfer situation. The significance level was set at 0.05. The Paleontological Statistics Software Package for Education and Data Analysis [30] was used to perform statistical analyses.

### Ethical considerations

The Swedish Ethical Review Authority (dnr 2018/2169- 31) reviewed and approved the study. The rights of the participants were carefully considered and protected in all aspects. Since the PwD lacked consent capability, the procedure of assent and dissent to participate was applied, as described by Black et al. [31], and in accordance with the Swedish Act concerning the ethical review of research involving humans [32]. It was carefully considered whether the PwD should be included in the study, based on the assent-dissent procedure, and consultation with the next of kin, the responsible nurse, and the caregivers who worked closest to the PwD. If there was uncertainty about what the person with dementia expressed or if the PwD was considered to be displeased or negatively affected, e.g. through increased anxiety or agitation the PwD was not included in the study. The PwD was asked for assent defined as “an affirmative agreement to participate as expressed verbally (i.e., orally) or a nonverbal indication of willingness to cooperate with study procedures” (31, p. 80) in close connection to each video taken of the person transfer situation. The information was adapted to the person's communicative ability, and the PwD was given time to respond to the oral information about video recording and study procedures. The person transfer situation was carefully observed by the first author, who is an experienced physiotherapist in dementia care, to detect any expression of dissent. Dissent is defined as “a verbal or non-verbal indication of unwillingness to participate in study procedures” (31, p. 81). Further, the assisting caregivers, who knew the person well, were asked if they detected any inconvenience of the PwD before, during and/or after the video recording. The first author, who video recorded, was at all times visible to the PwD, and no video recording was done in secret or hidden from the PwD. There was no video recording of the PwD undressed. Any verbal or nonverbal indication of unwillingness (i.e. dissent) during the observation would have led to immediate end of the video recording and exclusion of the PwD from the study. The first author contacted the next of kin via telephone, provided oral information about the study and the opportunity to ask questions and sent the written study information with the consent form by mail with a prepaid envelope. The next of kin could not make the decision on behalf of the PwD but rather could only state whether they did not oppose the PwD’s participation by responding to the question “Do you oppose the participation of your next of kin?”. The next of kin was asked, based on his or her experiences, how the PwD would have reacted to participate in a research study, before the development of late-stage dementia. The caregivers were provided with oral and written information of the study and asked to provide written and informed consent to participate.

## Results

### Care dyad 1

#### Step 4. Intervention planning and hypothesis testing

Based on the analysis from steps 1- 3 of the FBA, a tailored intervention was developed that focused on the nursing staff´s problematic transfer-related behaviour, DIDTAS items 9, 10, 11 and 14. The intervention was a combination of the following components: (1) the caregivers distributed the responsibility during person transfers so that one person maintained contact and interacted with the PwD and the other person pulled the sliding sheet and took care of the practicalities during the transfer; (2) the caregiver maintained eye contact and held the hands of the PwD during the turn, clearly informed what was going to happen and waited for a time between providing the information and performing the person transfer; (3) the turning was divided into several parts, namely, from back-lying to side-lying and from side-lying to back-lying; and (4) the PwD was prepared for movement by being positioned in a natural movement pattern for turning.

#### Step 5. Monitoring intervention effectiveness and modification

**Fig. 2:**
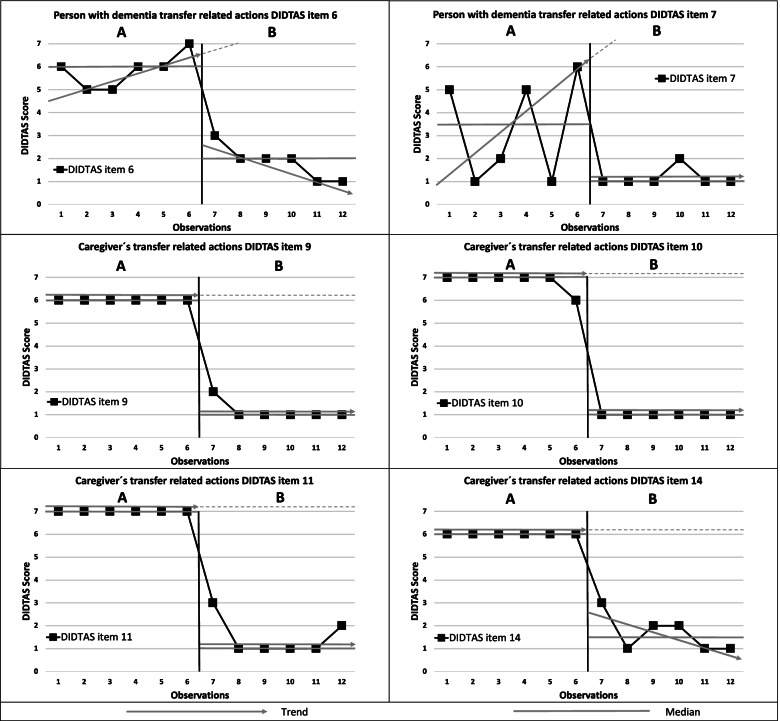
Data points, medians and phases for care dyad 1: DIDTAS item 6, PwD expresses discomfort through body language in the transfer situation; item 7, PwD expresses discomfort through words/sounds in the transfer situation; item 9, caregiver provides instructions for transfer just before beginning transfer; item 10, caregiver provides a clear verbal command about transfer; item 11, transfer request is followed by the caregiver waiting for the PwD to respond; and item 14, caregiver adapts their actions to facilitate the transfer situation of the PwD. A higher score indicated a more difficult person transfer situation

Visual inspection of DIDTAS items 6 and 7 showed a positive change in the trend. Verbal and nonverbal discomfort decreased in the intervention phase compared to the baseline [DIDTAS item 6: NAP = 100% (90% CI = 0.429- 1), *p* = 0.0039 and DIDTAS item 7: NAP = 79% (90% CI = 0.013- 1)]. The improvement in DIDTAS 6 showed low variability and short latency. The level between the baseline phase and intervention phase changed abruptly in DIDTAS 7 and DIDTAS 6. In DIDTAS 7 a high variability at baseline was observed, although stabilization could be seen in the intervention phase. Both DIDTAS item 6 and DIDTAS item 7 showed a median change (see Fig. 2).

**Fig. 3:**
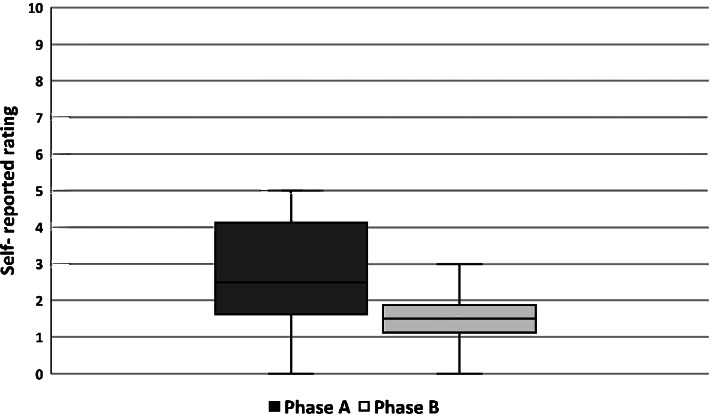
Caregivers’ self-reported ratings of perceived physical strain measured after the observation (where 0 = not physically strenuous at all and 10 = physically very strenuous). Each measurement point on which the box plot is based corresponds to the mean value of each self-reported rating from the two caregivers who participated in the transfer situation at the time of observation. Median phase A = 2.5; median phase B = 1.5

The caregivers’ self-reported ratings showed high self-efficacy, low catastrophizing thoughts and high perceived control for the transfer situation of interest. No change was observed for the caregivers’ self-reported ratings between the baseline and intervention phases, for further details, see additional file 2. The variability and median (median baseline = 2.5 and median intervention phase = 1.5) of the caregivers’ self-reported perceived physical strain decreased in the intervention phase, indicating that the caregiver experienced lower physical strain after the implementation of the intervention (see Fig. 3).

The caregivers´ performance improved in the intervention phase [DIDTAS 9, 10, 11 and 14: NAP = 100% (90% CI = 0.429- 1), *p* = 0.0039]. Caregiver trends changed positively as shown in DIDTAS items 9, 10, 11 and 14, indicating that the caregiver instructions and adaption of actions to facilitate the PwD improved. The variability for DIDTAS items 9, 10, 11 and 14 was low in the baseline phase and slightly higher in the intervention phase for DIDTAS 9, 11 and 14. The level changed rapidly between the baseline and intervention phases, and the latency was short after implementing the intervention for DIDTAS 9, 10, 11 and 14. The levels in phase B, as reflected by DIDTAS items 9, 10, 11 and 14, showed a median change (see Fig. 2).

The time required for the person transfer situation significantly increased (Mann–Whitney U-test, *p* = 0.008) between the baseline and intervention phases. The median increased from 57 s at baseline to 137 s in the intervention phase.

### Care dyad 2

#### Step 4. Intervention planning and hypothesis testing

According to the hypothesis, both the caregivers and the PwD displayed problematic transfer behaviour, as demonstrated by DIDTAS items 1 and 14, and the intervention to be prioritized was related to environmental factors. The intervention consisted of a combination of the following elements: (1) the PwD was helped to put on glasses before the person transfer began; (2) the left handle on the toilet was folded up before the PwD sat down, and then the left handle was folded down, and if necessary, the caregivers could lower the right handle during the person transfer; and (3) the caregivers provided support from the right side of the PwD.

#### Step 5. Monitoring intervention effectiveness and modification

**Fig. 4:**
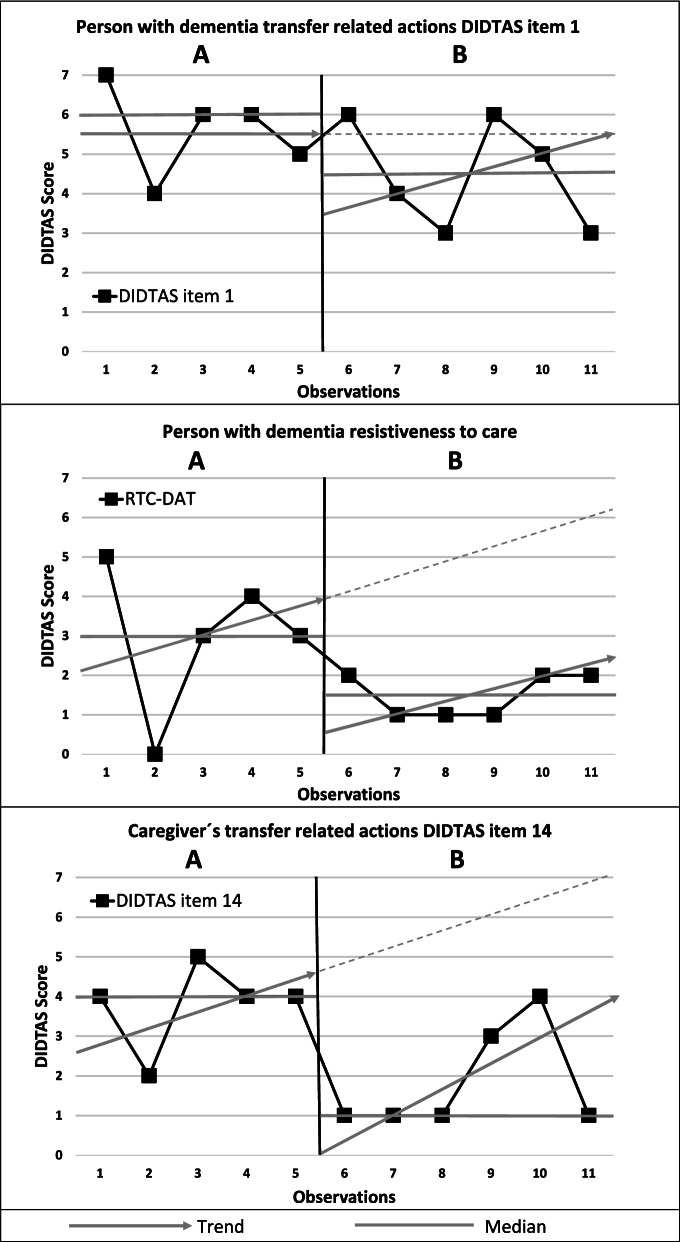
Data points, medians and phases for care dyad 2. DIDTAS item 1, the PwD is able to remain attentive during the transfer in the transfer situation; and DIDTAS item 14, caregiver adapts their actions to facilitate the transfer situation of the PwD. A higher score indicates a more difficult person transfer situation. For PwD resistiveness to care (RTC-DAT), a higher score indicates more resistiveness to care

In the intervention phase, the PwD was not capable of remaining attentive during the transfer situation, as reflected by an increasing trend in the intervention phase and no change in DIDTAS item 1 [DIDTAS item 1: NAP = 73% (90% CI = -0.134- 1)]. DIDTAS item 1 showed no distinct latency or shift in level, and the variability in both the baseline and intervention phases was high. DIDTAS item 1 showed a slight positive change in the median (see Fig. 4).

The PwD was less resistive to care in the intervention phase than the baseline phase [RTC-DAT: NAP = 80% (90% CI = -0.001- 1)]. The trend showed increases in both the baseline and intervention phases but at a lower level in the intervention phase. The RTC-DAT values showed a positive change in the median but did not show a distinct latency or shift in level. The variability was high in the baseline phase and intervention phase (see Fig. 4).

High self-efficacy, low catastrophizing thoughts, low perceived physical strain, and high perceived control in the transfer situation were reported by the caregivers. There was no change in self-reported ratings between the baseline and intervention phases, for further details, see additional file 2.

The caregivers improved their performance in the intervention phase [DIDTAS 14: NAP = 88% (90% CI = 0.166- 1], *p* = 0.0358]. The level between the baseline and intervention phases changed abruptly, and the latency was short. There was a change in the median between the baseline and intervention phases, and high variability was observed in both the baseline and the intervention phases (see Fig. 4).

The time required for the person transfer situation between the baseline and intervention phases did not change significantly. The median decreased from 171 s (2 min and 51 s) at baseline to 113 s (1 min and 53 s) in the intervention phase.

## Discussion

This study explored tailored interventions guided by an FBA for problematic person transfer situations in two dementia care dyads at an SCU. From a social cognitive theoretical perspective, the reciprocity between the individual, environmental and behavioural factors in the transfer situation was clearly illustrated in care dyad 1 but not as evident in care dyad 2.

In care dyad 1, nonverbal discomfort decreased (DIDTAS item 6) and a positive trend in both verbal and nonverbal discomfort (DIDTAS items 6 and 7) was observed. Caregivers’ transfer-related behaviour mirrored these changes by providing a clear verbal command (DIDTAS item 11) just before beginning the transfer (DIDTAS item 9), waiting for the PwD to respond (DIDTAS item 11) and adapting to the situation, such as keeping eye contact and holding the hands of the PwD during the transfer (DIDTAS item 14).

The high variability in DIDTAS item 7 in terms of addressing verbal discomfort in the baseline phase was stabilized in the intervention phase. It is well known that PwD behaviour can vary greatly [14], and stabilization, e.g., transfer-related behaviour, could imply an important improvement from a clinical perspective. Lack of time has been reported by caregivers as a barrier when providing palliative care for people with severe dementia [33]; however, in care dyad 1, the time for completing the person transfer increased by only approximately one minute. Simultaneously, the caregivers perceived less physical strain during the person transfer. The PwD in care dyad 1 suffered from paratonia, which is a motor behaviour problem prevalent in 90–100% of all PwDs with severe dementia and increases the caregiver burden with time [34]. Symptoms of paratonia in person transfers are crucial to address, and the intervention in care dyad 1, including caregivers’ verbal and nonverbal communication before initiating the transfer situation and waiting for the PwD to react, shows promising results for PwDs with paratonia.

In care dyad 2, high variability in PwD behaviour was seen in both the intervention and baseline phases. The transfer-related behaviour in the PwD was sustained despite the change in the caregivers’ behaviour, as reflected by their adaption of actions (DIDTAS item 14) and the inability of the PwD to remain attentive in the transfer situation (DIDTAS item 1). Possible reasons could be that the tailored intervention did not meet the needs of the PwD. The PwD tended to be less resistive to care in the intervention phase than the baseline phase. Reduced resistive behaviour was noted after a behavioural intervention in a previous study [6], which may indicate that these behaviours can be influenced by behavioural interventions.

In both care dyads 1 and 2, caregiver behaviour improved but still varied in the intervention phase. The variability may be related to the varying level of adherence to the intervention by the individual caregivers, which might reflect the difficulties for caregivers working in SCU to change their accustomed behaviour in their daily work. Caregiver training should focus on more intense and in-depth training to increase the understanding of behavioural change across the development of dementia [35]. Genuine professional knowledge concerning the fundamental needs in persons with dementia is recognized as crucial in caregivers for dyadic interactions in dementia care [36, 37], which means that health care professionals need to recognize the perspectives of both parts since the PwD needs to rely on the other’s contributions in the interaction [38].

There are both strengths and limitations in this study. The small number of cases and observations limits the generalizability, and the results should be interpreted in consideration of the piloting approach of the study. One limitation is that an AB design was used. Unfortunately, the health condition of the PwD made it impossible to add additional phases because the internal validity could have been strengthened by an ABAB design [19, 39]; however, the design was not feasible, mainly for two reasons. First, it would not have been ethical to end a caregiver behaviour after the intervention phase if it was beneficial for the PwD. Second, it would have been difficult for the caregiver to return to their former behaviour, although one possible change would have been to apply a follow-up phase [19]. In both care dyads, the intervention implemented was a combination of several components, and it might have been better to divide the intervention into several phases, such as an A-B1-B2-B3 sequence. Considering the need for interventions in both care dyads, such a division would have delayed the possibility for an improved transfer situation for the care dyad.

In care dyad 2, the scarce number of observations and high variability make it difficult to draw causal inferences about the impact of the intervention. More observations during a longer period might have better clarified the needs of the PwD and established a stable baseline. The FBA indicated that environmental factors affected the PwD behaviour in care dyad 2, but these factors were also influenced by personal factors, such as motor and social interaction behaviour [16]. The care dyad´s interaction is therefore complex, and the high variability complicated the analyses. It can take time for one factor to influence the others in the triad [16], and the intervention phase for care dyad 2 may be too short for this to happen. In care dyad 1, the variability in DIDTAS item 7 was high and a stable baseline was not achieved. An extended baseline and additional repeated observations would have been preferable to rule out the possibility that history or maturation could have influenced the change in the dependent variable [39, 40]. However, the rapid change of the PwD behaviour in care dyad 1 in the intervention phase together with the short latency in caregivers´ behavioural change indicates the impact of the intervention. Thunborg et al. [6] also demonstrated high variability and a short baseline in a care dyad, thus reflecting the challenges of conducting an intervention in this population. PwDs in the late phase of their dementia disease are frail, and their health condition can rapidly change. Such conditions increase the difficulty of following individuals over time, and in longitudinal dementia studies, dropouts due to death or other reasons commonly reduce the follow-up sample size [10]. For this reason, we tried to capture as many observations as possible during a short period of time. Unfortunately, only a portion of all the caregivers in the nursing home consented to participate in the study, and due to shift work, it was not possible to conduct and observe the care dyads every day in a row. On the other hand, this study reflects the reality of dementia care and the true conditions for the care dyad.

The results of the visual inspections were strengthened using NAP as a nonparametric statistical complement [29]. Because of high variability of data, trends could not be estimated with precision, and NAP is in such cases recommended as a suitable complement to visual inspection, to reduce the likelihood of misinterpretation [41]. However, NAP is only a way to “booster” the visual inspection in single case studies, and the results were well in line with the visual inspection.

Regarding the participants, especially the caregivers, we cannot rule out the possibility that testing could have influenced their actions during the patient transfer situations because they knew they were being filmed [39, 40]. The study-specific items used for the caregiver´s self-reported ratings were selected from valid and reliable instruments and self-efficacy items were formulated based on the recommendation by Bandura [25] to ensure good quality of question wording. These items were developed based on their relevance for the care dyads’ problems, and to our knowledge, no validated instrument addressed our specific research questions. The validity of the self-report items is therefore unknown. Further, we wanted to reduce the time needed for caregivers to fill in the questionnaires during their busy workday.

This study is a systematic replication of a former SCS by Thunborg et al. [6]. The present study was conducted in a new setting with regard to the nursing homes, caregivers and municipalities. Additionally, the constellation of the research team for this study was new. Although Thunborg’s participation in both studies could be considered a weakness, it aided in the precise operational definitions of DIDTAS.

Kazdin [42] emphasized that the selected participants in an SCS should be typical clinical cases. A strength of this study is that the two single cases illustrate two different person transfer situations commonly present in an SCU for PwD. Furthermore, the study was conducted in the participants´ natural environment. Every transfer situation was assessed with DIDTAS, PAINAID and RTC-DAT. The combination of these instruments meant that a behaviour could be evaluated from different aspects. For example, shouting can be interpreted as pain, a way to show resistance to care or an expression of discomfort with words/sounds. By carefully observing each behaviour with all three assessment scales, it was possible to form an idea of what function the behaviour had, which is an essential part in developing an FBA hypothesis [18].

It could be questioned if a PwD should be video- recorded during a personal care event, but it is important to improve difficult care situations for these vulnerable persons to augment their quality of life at the end stage of life. The impaired cognitive functioning of PwD entails ethical considerations and a high-level of expertise, and we made efforts to ensure not failing to respect the individuals with diminished consent capability. This was therefore carefully discussed with the Swedish Ethical Review Authority, who gave permission to the assent-dissent procedure as described by Black et al. [31].

## Conclusions

We could not demonstrate intrasubject replication in this study, but similar to the study by Thunborg et al. [6], our study showed promising results for one care dyad, which presented less discomfort expressed by the person with dementia and simultaneously improved the adaptive behaviour by the caregivers. The small number of cases and observations limits the generalizability, and the results should be interpreted in consideration of the piloting approach of the study.

The results indicate that the transfer-related behaviours of the care dyad might be improved through a behaviour-directed intervention tailored to meet the care dyad´s needs. Further research is needed to understand how behaviour interventions can be best constructed to meet the complexity of care dyads in problematic person transfer situations.

## Supplementary Information

**Additional file 1.** Dyadic interaction in dementia transfer assessment scale DIDTAS©.

**Additional file 2.** Caregiver´s Self-reported ratings. Medians and range (min-max) of the caregiver´s self-reported ratings. N= number of ratings of each self-report.

## Data Availability

The datasets generated and analysed during the current study are not publicly available due to ethical restrictions but are available from the corresponding author on reasonable request.

## Abbreviations

PwD: Person with Dementia
SCU: Special Care Unit
SCT: Social Cognitive Theory
SCS: Single Case Study
FBA: Functional Behavioural Analysis
DIDTAS: Dyadic Interaction in Dementia Transfer Assessment Scale
PAINAID: Pain Assessment in Advanced Dementia Scale
RTC-DAT: Resistiveness to Care Scale- Dementia Alzheimer Type
CI: Confidence Interval
NAP: Nonoverlap of All Pairs
