# Supplementary material for: Behaviour-directed interventions for problematic person transfer situations in two dementia care dyads: a single-case design study
Source: BMC Geriatr. 2022 Mar 29;22:261. doi: 10.1186/s12877-022-02952-5 (PMC8966167; doi:10.1186/s12877-022-02952-5)
Supplement: Supplementary file 1 — Additional file 1. Dyadic interaction in dementia transfer assessment scale DIDTAS©. [file 12877_2022_2952_MOESM1_ESM.pdf]

Additional file 1: Dyadic interaction in dementia transfer assessment scale DIDTAS©

Table S 1 Person with dementia transfer-related actions

**1/ Person with dementia is able to remain attentive in transfer situation**	Attentive			Partially attentive			Not attentive
	**1**	**2**	**3**	**4**	**5**	**6**	**7**
**2 /Person with dementia is able to actively participate in the transfer situation**	Active participation			Partially active participation			No active participation
	**1**	**2**	**3**	**4**	**5**	**6**	**7**
**3/ Person with dementia has goal-orientated movement pattern in transfer situation**	Objective movement pattern			Partially goal-orientated movement pattern			No goal-orientated movement pattern
	**1**	**2**	**3**	**4**	**5**	**6**	**7**
**4/ Person with dementia moves at a goal-orientated tempo**	Goal-orientated tempo			Partial goal-orientated tempo			No goal-orientated tempo
	**1**	**2**	**3**	**4**	**5**	**6**	**7**
**5 /Person with dementia has bodily control in relation to his/her surroundings**	Complete control			Partial control			No control
	**1**	**2**	**3**	**4**	**5**	**6**	**7**
**6/ Person with dementia does not express discomfort through body language in the transfer situation**	No discomfort expressed through body language			Partial discomfort expressed through body language			Discomfort expressed through body language
	**1**	**2**	**3**	**4**	**5**	**6**	**7**
**7/ Person with dementia does not express discomfort through words/sounds in the transfer situation**	No discomfort expressed with words/sounds			Partial discomfort expressed with words/sounds			Discomfort expressed with words/sounds
	**1**	**2**	**3**	**4**	**5**	**6**	**7**
**8/ Person with dementia is independent in transfer situation**	Independent			Partially independent			Not independent
	**1**	**2**	**3**	**4**	**5**	**6**	**7**

Table S 2 Caregiver's transfer-related actions

**9/ Caregiver provides instructions for transfer just before beginning transfer**	Instructions is given just before start			Limited instructions given just before start			No instructions is given just before start
	1	2	3	4	5	6	7
**10/ Caregiver provides a clear verbal command about transfer**	Command is clear			Command is partially clear			Command is unclear
	1	2	3	4	5	6	7
**11/Request for transfer is followed by the caregiver waiting for the Person with dementia to respond**	Caregiver waits during Person with dementia transfer			Caregiver partially waits during Person with dementia transfer			Caregiver does not wait during Person with dementia transfer
	1	2	3	4	5	6	7
**12/ If two caregivers are present, one of them assists with cooperation of the Person with dementia** ☐ **Not applicable**	Cooperation controlled by one caregiver			Cooperation partially controlled by one caregiver			Cooperation not controlled by one caregiver
	1	2	3	4	5	6	7
**13/ Transfer situations are performed in a safe manner for the Person with dementia**	Transfer situation is safe			Transfer situation is partially safe			Transfer situation is not safe
	1	2	3	4	5	6	7
**14/ Caregiver adapts their actions to facilitate the transfer situation of the Person with dementia**	Actions are adapted			Actions are partially adapted			Actions are not adapted
	1	2	3	4	5	6	7
**15/ Caregiver maintains contact with the Person with dementia during the transfer situation**	Contact maintained			Contact partially maintained			Contact not maintained
	1	2	3	4	5	6	7
**16/ Transfer aids available before start of transfer situation**	Transfer aids available			Transfer aids partially available			Transfer aids not available
	1	2	3	4	5	6	7
**17/ Interaction with the Person with dementia is optimal for the transfer situation**	Interaction is optimal			Transfer situation partially optimal			Interaction is not optimal
	1	2	3	4	5	6	7
