# Supplementary material for: Behaviour-directed interventions for problematic person transfer situations in two dementia care dyads: a single-case design study
Source: BMC Geriatr. 2022 Mar 29;22:261. doi: 10.1186/s12877-022-02952-5 (PMC8966167; doi:10.1186/s12877-022-02952-5)
Supplement: Supplementary file 2 — Additional file 2. Caregiver´s Self-reported ratings. Medians and range (min-max) of the caregiver´s self-reported ratings. N= number of ratings of each self-report. [file 12877_2022_2952_MOESM2_ESM.docx]

**Additional file 2: Caregiver´s Self-reported ratings**

Medians and range (min-max) of the caregiver´s self-reported ratings. N = number of ratings of each self-report.

|  | **Care dyad 1** | | **Care dyad 2** | |
| --- | --- | --- | --- | --- |
| **Self- reported ratings** | **Phase A**  median  (min - max) | **Phase B**  median  (min - max) | **Phase A**  median  (min - max) | **Phase B**  median  (min - max) |
| 1) How confident are you about your ability to carry out the transfer (0 = not at all confident, 10 = completely confident) | 10 (N = 12)  (10 - 10) | 10 (N = 12)  (9 - 10) | 9 (N = 5)  (5 - 9) | 8.5 (N = 4)  (6 - 9) |
| 2/ How confident are you about your ability to manage the transfer even when it is the most problematic? (0 = not at all confident, 10 = completely confident) | 9 (N = 12)  (9 - 10) | 9 (N = 12)  (9 - 10) | 8 (N = 5)  (6 - 9) | 8 (N = 4)  (7 - 9) |
| 3) How confident are you about your ability to carry out the transfer even if a sudden disruptive moment occurs? (0 = not at all confident, 10 = completely confident) | 9 (N = 12)  (7 - 10) | 9 (N = 12)  (9 - 10) | 8 (N = 5)  (5 - 10) | 8 (N = 4)  (5 - 10) |
| 4) How confident are you about your ability to carry out the transfer even when you feel stressed? (0 = not at all confident, 10 = completely confident) | 9.5 (N = 12)  (8 - 10) | 9 (N = 12)  (9 - 10) | 10 (N = 5)  (9 - 10) | 8.5 (N = 4)  (7 - 10) |
| 5/ The transfer is awful, and I feel it is impossibly difficult to carry it out. (0 = do not agree at all, 10 = fully agree) | 1 (N = 12)  (0 - 1) | 1 (N = 12)  (0 - 2) | 1 (N = 5)  (0 - 2) | 2 (N = 4)  (1 - 5) |
| 6) Considering what you do during an average workday to manage the transfer: Which control do you perceive that you have over the transfer situation? (0 = not control at all, 6 = complete control) | 4.5 (N = 12)  (3 - 6) | 5.5 (N = 12)  (5 - 6) | 3 (N = 5)  (1 - 6) | 3.5 (N = 4)  (2 - 5) |
| 7) Considering what you do during an average workday to manage the transfer: To what degree is it possible for you to manage the difficulties in the transfer situation? (0 = not at all possible at all, 6 = completely possible) | 6 (N = 12)  (5 - 6) | 5 (N = 12)  (5 - 6) | 3 (N = 5)  (1 - 6) | 3.5 (N = 4)  (2 - 5) |
| 8) How do you experience the physical strain during the person transfer situation (0 = not physically strenuous at all, 10 physically very strenuous) | 2.5 (N = 12)  (0 - 7) | 1.5 (N = 12)  (0 - 3) | 2 (N = 5)  (1 - 7) | 2 (N = 5)  (1 - 3) |
